# The role of epidermal sphingolipids in dermatologic diseases

**DOI:** 10.1186/s12944-016-0178-7

**Published:** 2016-01-19

**Authors:** Sonia Borodzicz, Lidia Rudnicka, Dagmara Mirowska-Guzel, Agnieszka Cudnoch-Jedrzejewska

**Affiliations:** Department of Experimental and Clinical Physiology, Laboratory of Centre for Preclinical Research, Medical University of Warsaw, Banacha 1B, 02-097 Warsaw, Poland; Department of Dermatology, Medical University of Warsaw, Koszykowa 82A, 02-008 Warsaw, Poland; Department of Experimental and Clinical Pharmacology, Laboratory of Centre for Preclinical Research, Medical University of Warsaw, Banacha 1B, 02-097 Warsaw, Poland

**Keywords:** Sphingolipids, Ceramide, Sphingosine-1-phosphate, Dermatologic diseases, Psoriasis, Atopic dermatitis, Ichthyosis

## Abstract

Sphingolipids, a group of lipids containing the sphingoid base, have both structural and biological functions in human epidermis. Ceramides, as a part of extracellular lipids in the stratum corneum, are important elements of the skin barrier and are involved in the prevention of transepidermal water loss. In addition, ceramides regulate such processes as proliferation, differentiation and apoptosis of keratinocytes. Another important sphingolipid, sphingosine-1-phosphate (S1P), inhibits proliferation and induces differentiation of keratinocytes. A recent clinical study of the efficacy and safety of ponesimod (a selective modulator of the S1P receptor 1) suggested that sphingolipid metabolism may become a new target for the pharmacological treatment of psoriasis. The role of sphingolipids in some dermatologic diseases, including psoriasis, atopic dermatitis and ichthyoses was summarized in this article.

## Background

Sphingolipids have both structural and biological functions in human epidermis [1, 2]. The barrier between the environment and the human body is maintained by the stratum corneum (SC), the most external layer of the epidermis. The stratum corneum is composed of terminally differentiated keratinocytes and extracellular lipids, such as ceramides (CER), cholesterol and free fatty acids in almost equimolar quantities. The main function of these lipids in the stratum corneum is the formation of the skin barrier and the prevention of transepidermal water loss (TEWL) [1, 3]. Ceramides are among the most important epidermal sphingolipids and compose about 50 % of intercellular stratum corneum lipids by mass [3]. The stratum corneum sphingolipid metabolism has been studied in many dermatologic diseases, such as psoriasis [4–6], atopic dermatitis (AD) [7–9], hand eczema [10], acne vulgaris [11], autosomal recessive congenital ichthyosis [12, 13] including harlequin ichthyosis [14] and lamellar ichthyosis [15, 16], bullous ichthyosiform erythroderma [16–18], keratitis-ichthyosis-deafness syndrome [19], Dorfman-Chanarin syndrome [20], Netherton syndrome [21], Sjögren-Larsson syndrome [16], hypohidrotic ectodermal dysplasia [22], Gaucher disease [23] and Niemann-Pick disease [24].

In this article, the role of sphingolipids in dermatologic diseases was summarized.

## General characteristics of epidermal sphingolipids – structure, synthesis and function

**Fig. 1:**
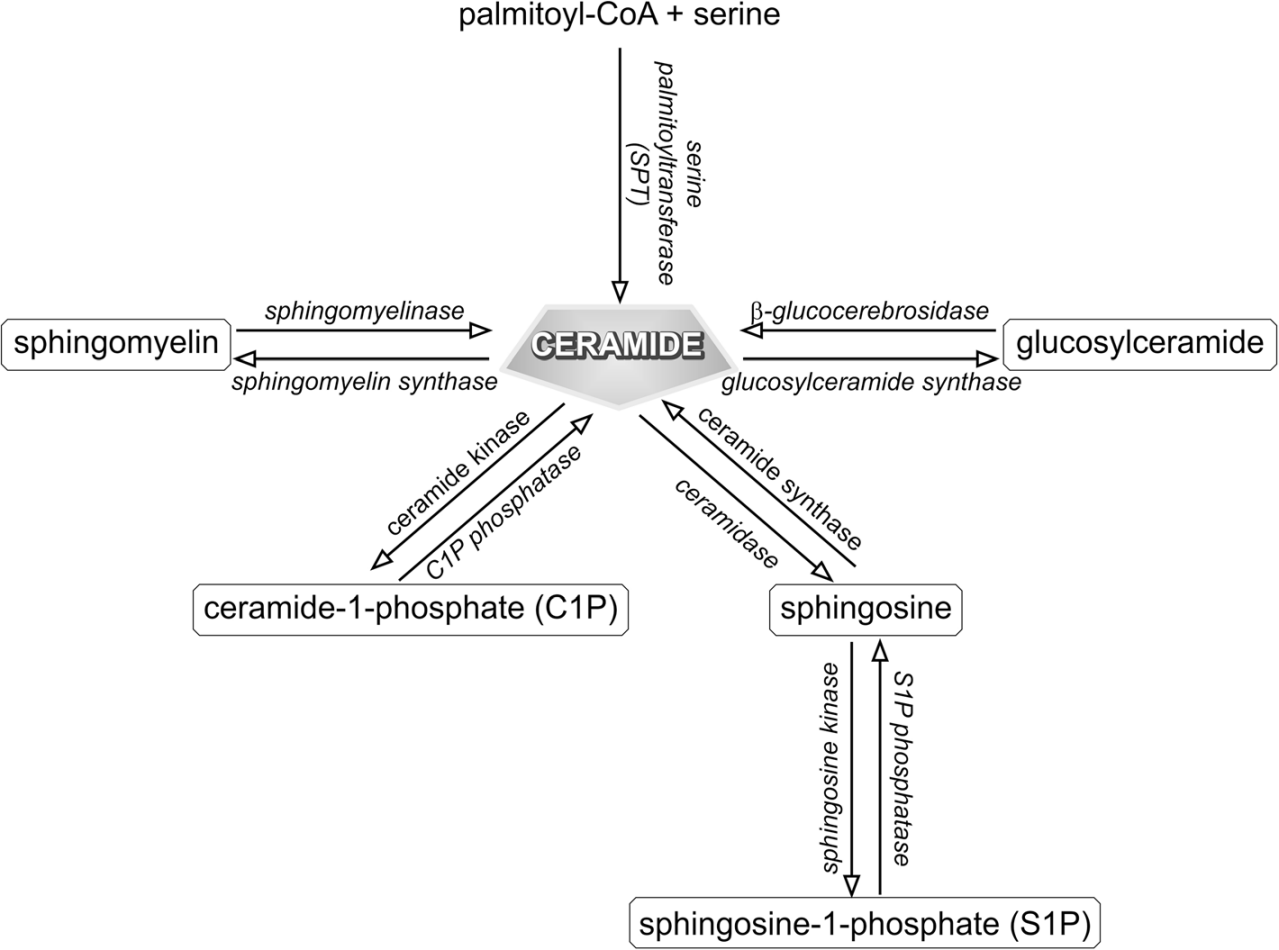
Pathways of sphingolipid metabolism

Sphingolipids are a group of lipids containing the sphingoid base, which is formed by the condensation of an amino acid and a fatty acid. The sphingoid bases are enzymatically modified to generate a wide range of biologically active sphingolipids, including ceramides, sphingomyelin, sphingosine-1-phosphate (S1P), ceramide-1-phosphate, and glycosphingolipids (Fig. 1) [25, 26].

The basic structure of the main epidermal sphingolipids, ceramides, is a sphingoid base with fatty acid connected by an amide bond. Four types of fatty acids (esterified ω-hydroxy [EO], ω-hydroxy [O], α-hydroxy [A] and non-hydroxy [N] fatty acids) with four types of sphingoid bases (sphingosine [S], 6-hydroxysphingosine [H], dihydrosphingosine [DS] and phytosphingosine [P]) create 16 classes of ceramides, which have been identified in stratum corneum [27]. Ceramides species are differentiated by the length of the fatty acid or the sphingoid backbone. There are three pathways of ceramide generation in the skin: 1) de novo synthesis in the endoplasmic reticulum via serine palmitoyltransferase (SPT); 2) degradation of glucosylceramides by β-glucocerebrosidase; and 3) hydrolysis of sphingomyelin by sphingomyelinase [28, 29]. Ceramides synthesized in the endoplasmic reticulum are moved to the Golgi apparatus, where the transformation to glucosylceramides or sphingomyelin occurs. Next, these substances are transported from the Golgi apparatus into secretory vesicles – epidermal lamellar bodies, which fuse with the plasma membrane when the keratinocytes cross the line between the stratum granulosum and stratum corneum. In the intercellular space of the stratum corneum, glucosylceramides and sphingomyelin are converted back to ceramides via hydrolysis by β-glucocerebrosidase and sphingomyelinase, respectively [3, 29]. Ceramides may be modified to other biologically active sphingolipids, for example, sphingosine, which is phosphorylated to sphingosine-1-phosphate by sphingosine kinase and sphingomyelin, which is converted to sphingosylphosphorylcholine via sphingomyelin deacylase [2, 30].

Other than the structural function in the epidermis, sphingolipids also play an important role in epidermal signaling. Ceramides regulate such processes as proliferation, differentiation and apoptosis [31]. In vitro studies performed in normal cultured human keratinocytes revealed that ceramides activated apoptosis signal-regulating kinase 1 (ASK1) and stimulated production of caspase-14, which both are importantly involved in the differentiation of keratinocytes [32, 33]. Ceramides also induced apoptosis of cultured human keratinocytes triggered by ultraviolet B irradiation [34, 35]. Synthetic short-chain analogues of ceramides inhibited proliferation and induced differentiation of the human squamous cell carcinoma cell line – DJM-1 [36]. Mice deficient of UDP-glucose ceramide glucosyltransferase (UGCG), which glucosylates ceramides in the Golgi apparatus, showed an ichthyosis-like skin phenotype with impaired differentiation of keratinocytes [37]. In most cell types, S1P has antiapoptotic and mitogenic properties, acting as a ceramides antagonist [30]. Interestingly, in human cultured keratinocytes, S1P inhibited proliferation and induced their differentiation, however, it did not promote apoptosis [38]. Human keratinocytes express all known types of S1P receptors – S1P_1_-S1P_5_ [38]. It has been suggested that keratinocyte growth inhibition induced by S1P is mediated by inactivation of the Akt/protein kinase B (PKB) pathway [39]. Moreover, in cultured human keratinocytes, S1P reduced the synthesis of cyclin D2 (an activator of cell cycle) and increased levels of inhibitors of cyclin-dependent kinases p21 and p27 (involved in cell cycle arrest) [39].

In vitro investigation revealed that sphingosylphosphorylcholine added to the cultures of human keratinocytes enhanced their proliferation and promoted wound repair by stimulating migration and/or proliferation of the keratinocytes to the denuded areas of keratinocytes monolayers. The cutaneous wound healing properties of sphingosylphosphorylcholine was also confirmed in vivo in genetically healing-impaired diabetic (db/db) mice [40].

## Sphingolipids in skin diseases

### Psoriasis

In vivo animal studies confirm the essential role of ceramides in the pathogenesis of psoriasis. Nakajima et al. [41] reported that newborn SPT- knockout mice showed generalized xerosis, had significantly decreased epidermal levels of ceramide and had significantly impaired water-holding capacity compared to wild type mice. The transepidermal water loss was normal at birth, but in 3-day-old SPT-knockout mice the transepidermal water loss after tape stripping (a mechanical irritation of the skin which disrupts the skin barrier function) significantly increased compared to wild type mice. Two-week-old and older mice lacking SPT presented psoriasis-like skin lesions with histologically confirmed hyperkeratosis, acanthosis, loss of the granular layer and infiltration of inflammatory cells, including neutrophils in the upper parts of the dermis and aggregation of neutrophils similar to “the Munro’s microabscess”. Both the psoriatic-like skin lesions and draining lymph nodes had elevated levels of γδ T cells producing interleukin-17 (IL-17), and most of them producing also interleukin-22 (IL-22). Moreover, CD11c^+^ cells producing interleukin-23 (IL-23) were also found in psoriatic-like skin lesions. The authors of the study suggest that epidermal depletion of ceramide levels was responsible for the formation of psoriasis-like skin lesions and this effect was mediated by IL-23-dependent γδ T cells which also produce IL-22, although the exact mechanism still needs to be determined [41].

The studies performed in skin biopsies taken from patients with psoriasis revealed that the level of de novo ceramides synthesis, the protein expression of SPT and the amount of ceramides are significantly lower in psoriatic plaques compared to the non-lesional epidermis [4–6]. Moreover, the percentage reductions of both – ceramide synthesis and its epidermal level – were positively correlated with the Psoriasis Area and Severity Index (PASI) score in mild to moderate psoriasis [4, 5]. Interestingly, the level of apoptotic signaling molecules, such as protein kinase C-alpha (PKC-α) and c-jun N-terminal kinase (JNK) were significantly reduced in lesional epidermis in comparison to non-lesional epidermis. Presumably, the reduction in the ceramide levels in psoriatic lesions had an antiapoptotic effect with concomitant enhancement of epidermal proliferation [4].

Not only the total amount but also the composition of ceramides is changed in psoriatic skin.

Psoriatic lesions had an altered distribution of ceramides, for example, a significantly decreased amount of ceramide 1 (CER[EOS] – ceramides containing esterified ω-hydroxy fatty acid with sphingosine) in comparison to healthy skin. The alteration of ceramide composition in psoriatic plaques was associated with a significantly increased level of transepidermal water loss [42]. Another study performed by Motta et al. [43] showed that psoriatic plaques had decreased levels not only of CER[EOS] but also of ceramides containing phytosphingosine with a concomitant elevated concentration of ceramides containing sphingosine in comparison to normal healthy skin. However, in this study, the total amount of ceramides in psoriatic lesions was unchanged in comparison to healthy skin. Tawada et al. [44] observed that psoriatic lesions have a significantly lower proportion of ceramides containing long-chain fatty acids in comparison to healthy skin. The authors of the study also reported that interferon γ, which had been previously reported to be abundant in psoriatic skin, reduced the mRNA expression of ELOVL (elongase of long-chain fatty acids) and ceramide synthase in cultured human keratinocytes. Since both of these enzymes are responsible for elongation of the chain of fatty acids, the authors suggest that this observation may explain the reduction of ceramides containing long-chain fatty acids in psoriatic skin [44].

There are several hypotheses which might explain the observed reduction in the amount of ceramides in human psoriatic skin. Psoriatic non-lesional skin had decreased mRNA expression of glucosylceramide-β-glucosidase compared to normal healthy skin, although the mRNA level of this enzyme was higher in psoriatic plaques than in non-lesional skin [28]. Psoriatic lesions (both active-type and chronic-type plaques) had also significantly decreased protein levels of prosaposin in comparison to psoriatic non-lesional skin and healthy skin. Prosaposin is a precursor of saposin, a nonenzymatic cofactor, which is required in the hydrolysis of sphingolipids, for example, by β-glucocerebrosidase. Therefore, reduced levels of prosaposin and saposin in psoriatic skin may disturb the enzymatic transformation of glucosylceramides to ceramides [45]. Moreover, the level of another enzyme involved in ceramide generation (sphingomyelinase) was decreased in the stratum corneum of psoriatic lesions compared to non-lesional psoriatic skin [45].

Not only ceramides but also other sphingolipid levels are altered in psoriatic skin.

In human psoriatic lesions, the levels of sphingoid bases – sphingosine and dihydrosphingosine (sphinganine) – were significantly higher in comparison to non-lesional skin. Also, a significant positive correlation was observed between the percentage change of ceramidase protein expression and the PASI score [46].

The analysis of skin biopsies taken from psoriatic patients revealed that mRNA expression of S1P phosphatase 2 was significantly increased in psoriatic lesions compared to non-lesional skin, however, there was no significant alteration in the expression levels of S1P phosphatase 1 and sphingosine kinases, both type 1 and 2. Since many inflammatory stimuli enhance the expression and activity of SPP2, the authors of the study suggest that SPP2 may be involved in inflammatory signaling and probably plays a role in the pathogenesis of psoriasis [47].

Owing to the fact that psoriatic skin shows disturbances in sphingolipid structure in comparison to healthy skin and deficiency of SPT, an enzyme essential in ceramide synthesis, leads to formation of psoriatic-like lesions in mice, it may be assumed, that new drugs which restore the physiological sphingolipid metabolism may become an important way for the pharmacological treatment of psoriasis.

#### Ponesimod as the novel therapeutic agent for patients with psoriasis

Ponesimod is a selective, reversible, orally active modulator of S1PR_1_ (S1P receptor 1), which causes internalization of S1PR_1_ resulting in the desensitization of T and B cells to the gradient of S1P concentration and therefore diminishes lymphocytes exit from secondary lymphoid organs with concomitant reduction of circulating lymphocytes count [48, 49].

A double-blind, randomised, placebo-controlled, parallel-group, multicentre phase 2 study of the efficacy, safety and tolerability of oral ponesimod in patients with moderate to severe chronic plaque psoriasis was performed [48]. In week 16 of the treatment, 46 % of 126 patients who received 20 mg of ponesimod daily presented a 75 % reduction in PASI score and doubling the dose improved the efficacy to the 48.1 % (in a group of 133 patients), whereas the placebo group reached the endpoint in only 13.4 % of the patients. The most common adverse events were: dyspnea, serum transaminases elevation, headache, nasopharyngitis, dizziness, bradycardia, pruritus and coughing, whereas the most frequent adverse events which required the termination of the study were: dyspnea, wheezing, second-degree atrioventricular block and elevation of the serum hepatic enzyme level [48]. As ponesimod dose-dependently reduces the total lymphocyte count, the authors of the study suggest that a beneficial outcome of the treatment may result from the sequestration of lymphocytes in the lymph nodes which therefore suppress the formation of psoriatic lesion [48]. The results of the above study suggest that ponesimod might be a first in this class oral therapeutic for psoriasis [50]. According to its anti-inflammatory properties, it is also extensively studied for another autoimmune disease, namely, multiple sclerosis.

### Atopic dermatitis

The mouse model of atopic dermatitis induced with cutaneous applications of 4-ethoxymethylene-2-phenyl-2-oxazolin-5-one revealed decreased expression and protein levels of ELOVL 1 and ELOVL 4, which are essential elongases for the production of very long-chain fatty acids and are importantly involved in maintaining the skin barrier function [51, 52].

Human studies reported that the stratum corneum ceramide composition in AD lesions is altered in comparison to healthy skin and also diminished levels of total ceramide count in AD skin was reported [7–9]. Also, within several classes of ceramides, human skin affected by AD showed decreased levels of larger ceramide species and elevated levels of smaller ceramides and that observation has also been confirmed in the mouse model of AD [8, 51, 53]. The skin of patients with atopic eczema also had a reduced ceramide/cholesterol ratio [54]. In comparison to healthy skin, both affected and non-lesional skin from patients with AD had a significantly reduced amount of ω-hydroxyceramides, which are connected to the epidermal cornified envelope and are importantly involved in maintaining the epidermal barrier function [55].

In non-lesional AD skin compared to healthy skin, the statistically significant reduction of ceramide levels was observed in some studies [7, 56], while others [57, 58] did not show any statistically significant changes between those two investigated groups. Farawanah et al. [57] reported that an analysis of ceramide classes between uninvolved AD and healthy skin did not show significant changes but Bleck et al. [59] showed a significant change in the composition of several ceramide classes in non-eczematous skin of atopic eczema [57, 59]. However, a significant difference was observed in the ceramide chain length. The level of ceramides with an extremely short chain length was markedly increased in several ceramide classes, while the concentration of very long-chain ceramides containing esterified ω-hydroxy fatty acid with phytosphingosine (CER[EOP]) or 6-hydroxysphingosine (CER[EOH]) was significantly decreased in non-lesional skin of patients with atopic eczema [58].

The reduction of ceramide chain length in non-lesional skin was also correlated with increased transepidermal water loss, altered intercellular lipid organization and disease severity, but did not correlate with filaggrin mutation genotypes, which is believed to be strongly associated with the pathogenesis of AD [58].

Some studies, which analyze ceramide composition, divide AD patients into two groups: filaggrin mutation carriers and non-filaggrin AD patients. Angelova-Fischer et al. [60] reported that non-lesional skin of AD filaggrin mutation carriers had a decreased ceramide/cholesterol ratio in comparison to the non-filaggrin AD type and healthy controls. Moreover, in AD lesions filaggrin mutation carriers, the amount of CER[EOH] was significantly reduced in comparison to the non-filaggrin AD type. In another study, a significant reduction of this class of ceramides was observed in non-lesional atopic eczema skin compared to the healthy controls with and without filaggrin mutation [60, 61].

Several hypotheses might explain the cause of ceramide deficiency observed in the stratum corneum of AD patients. It is suggested that the activity of sphingomyelin glucosylceramide deacylase, an enzyme which deacylates glucosylceramide or sphingomyelin to glucosylsphingosine and sphingosylphosphorylcholine respectively instead of to ceramide, leads to the depletion of ceramide and altered barrier function in AD skin [62]. Between atopic uninvolved and healthy control skin samples, there were no significant differences in the activity of β-glucocerebrosidase and ceramidase [63]. The activity of bacterial ceramidase in the flora of the skin of patients with AD is suggested as another possible cause of ceramide deficiency in AD skin [64, 65]. Also, both lesional and non-lesional skin from patients with AD had significantly decreased epidermal acid and neutral sphingomyelinase activity compared to the controls [66]. The lower activity of sphingomyelinases may result from a reduced protein level of prosaposin, which was observed in non-lesional AD skin at a statistically significant level [67]. As the Th1 and Th2 immune responses had been reported to play an important role in the pathogenesis of AD, Sawada et al. [68] suggested that a reduction of ceramides in AD skin may be the result of the Th2 type of inflammation. Administration of Th2 cytokines, interleukin-4 (IL-4) and interleukin-6 (IL-6), to the reconstructed human epidermal keratinization model significantly reduced the stratum corneum levels of ceramide with concomitant reduction of the SPT-2, acid sphingomyelinase and β-glucocerebrosidase expression in the epidermis, while the addition of Th1 cytokines, granulocyte-macrophage colony-stimulating factor (GM-CSF), interferon-γ (IFN-γ) and tumor necrosis factor α (TNF-α), increased the levels of ceramide, which was not correlated with any alteration of enzyme expression [68]. Tawada et al. [44] suggested that IFN-γ may be importantly involved in the reduction of ceramides containing long chain fatty acids in AD skin, in a similar way as in psoriasis as described above.

It was observed that both involved and uninvolved AD skin had decreased levels of sphingosine and reduced activity of acid ceramidase with a concomitant increased number of bacteria including Staphylococcus aureus. As sphingosine has antimicrobial properties and plays a role in antibacterial protection in healthy skin, the authors of the study suggest that colonization of bacteria in the AD skin was correlated with decreased levels of sphingosine, which resulted from reduced levels of the substrate – ceramides and diminished activity of acid ceramidase, the enzyme involved in the production of sphingosine [69].

Results of the studies mentioned above suggest that pharmacological normalization of altered sphingolipid composition in AD skin may restore the impaired skin barrier function, prevent excess transepidermal water loss and diminish skin bacterial colonization in patients with atopic dermatitis.

### Ichthyoses

The three types of autosomal recessive congenital ichthyoses, including harlequin ichthyosis, lamellar ichthyosis and congenital ichthyosiform erythroderma are caused by a mutation in the ATP-binding cassette transporter A12 (ABCA12) gene [70]. ABCA12 is a transmembrane transporter, involved in the transport of lipids in lamellar bodies to the upmost surface of epidermal stratum granulosum [70]. The human cultured keratinocytes obtained from patients with harlequin ichthyosis carrying a mutation of ABCA12 showed glucosylceramide accumulated around the nuclei, unable to reach the external regions of the cytoplasm [14]. Moreover, the epidermis of these patients presented dispersed localization of glucosylceramide, while in healthy skin the distribution was narrowed [14]. In mice, ABCA12 deficiency was associated with very similar observations and also with a significant reduction in total ceramide levels, especially of CER[EOS] and a significant increase in their glucosylceramide precursors, however, the ceramide composition and distribution was supposed to normalize during maturation of ABCA12 skin [71, 72].

Patients with autosomal recessive lamellar ichthyosis had significantly increased TEWL, different relative concentrations of several ceramide fractions and free fatty acid-ceramide ratio in comparison to the healthy controls [15]. Moreover, patients with non-erythrodermic lamellar ichthyosis had a reduced level of ceramide 1 in comparison to the healthy skin, whereas cases of limited lamellar ichthyosis did not show any significant changes in the amount of ceramide 1 [16].

Patients with autosomal recessive congenital ichthyosis presented the homozygous mutation of CERS3 gene, encoding the ceramide synthase three enzyme, which was associated with abnormal ceramide composition (for example, reduced levels of very long-chain ceramides) and disturbed differentiation of keratinocytes [12, 13].

Autosomal recessive congenital ichthyosis is also triggered by the mutations in ALOX12B or ALOXE3 genes, encoding the lipoxygenases 12R-LOX and epidermal LOX-3, respectively, which are both involved in the transformation of fatty acid substrates to epoxy alcohol derivatives and play an important role in the barrier function of epidermis. eLOX-3 and 12R-LOX deficient mice, which died a few hours after birth, had significantly increased TEWL and altered ceramide skin composition in comparison to wild type mice [73, 74].

In the animal model of bullous ichthyosiform erythroderma (also known as epidermolytic hyperkeratosis), both homozygous and heterozygous mice lacking keratin-10 compared to wild type mice presented significantly increased levels of ceramide 2 (CER[NS]) with a concomitant reduction of several other classes of ceramides to the total amount of SC lipids, and also reduced levels of sphingomyelin and glucosylceramide [17]. Homozygous and heterozygous newborn mice lacking keratin-10 had significantly increased TEWL, however, in adult heterozygous mice the TEWL was significantly lower in comparison to wild type mice [18]. The activity of neutral sphingomyelinase was significantly increased in homozygous and heterozygous keratin-10 deficient mice, with significantly decreased activity of acid sphingomyelinase, which may explain the observed relative decrease of some ceramide classes to the total amount of SC lipids [18]. It has been documented that human stratum corneum total ceramide levels were reduced in bullous ichthyosiform erythroderma [16].

Heterozygous Cx26S17F mice with a mutation in the gap junctional channel protein connexin 26 presented characteristic features of human keratitis-ichthyosis-deafness syndrome. Recently, Bosen et al. [19] have reported that the skin of these animals compared to the control mice presented hyperproliferation and hyperkeratosis, both of which were observed only in adult mice, secretion of lipids in the stratum granulosum and a significant decrease of CER[EOS] in the external epidermal surface, but only slight in the whole epidermis of newborn mice. The authors suggest that this type of ceramide was synthesized properly, but the secretion of these lipids to the epidermal surface was impaired, which may have a negative impact on the epidermal barrier function [19].

Dorfman-Chanarin syndrome is an autosomal recessive disease with concomitant ichthyosis in human skin. It was observed that the SC from patients with this syndrome had decreased levels of unbound acylceramides as well as a reduced amount of bound ω-hydroxy ceramides which forms the keratinocyte-bound lipid envelope, important in maintaining the skin barrier function [20].

### Other skin diseases

Netherton syndrome is an autosomal recessive skin disorder which causes such symptoms as erythroderma, a hair shaft defect (trichorrhexis invaginata) and constant atopic manifestations [21, 75]. The stratum corneum of patients with Netherton syndrome presented significantly increased levels of short-chain ceramides and some of the patients had reduced levels of acylceramides [21].

Sjögren-Larsson syndrome is an autosomal recessive disease resulting from mutations in the fatty aldehyde dehydrogenase gene [76]. The stratum corneum of patients with Sjögren-Larsson syndrome presented decreased levels of ceramide 1, 6 and 7 [16; 76]. Moreover, there was a marked elevation in the amount of membrane-bound ceramides forming the keratinocyte-bound lipid envelope [76]. Interestingly, the TEWL of the Sjögren-Larsson syndrome skin lesion was not changed, although the effectiveness of water retention was decreased [76].

Hypohidrotic ectodermal dysplasia is a genetic disease, characterized by the inability to sweat and atopic dermatitis-like skin lesions [22]. Comparison of the SC lipids between patients with hypohidrotic ectodermal dysplasia and atopic dermatitis revealed that hypohidrotic ectodermal dysplasia was associated with significantly increased levels of CER[EOS], with no concomitant alteration in other ceramide classes [22].

Gaucher disease is a genetic disease caused by a deficiency in the lysosomal enzyme β-glucocerebrosidase gene and has three subtypes categorized by the existence of neurological symptoms [77, 78]. The majority of patients with Gaucher disease type 2 do not have skin alterations, however, some patients presented ichthyosiform skin [23]. Epidermis from β-glucocerebrosidase deficient mice showed accumulation of glucosylceramides with concomitant reduction of the related ceramides and significantly increased TEWL in comparison to wild type mice [78–80]. Studies performed in patients with Gaucher disease type 1, 2 and 3 revealed that only epidermis from patients with Gaucher disease type 2 presented an elevated ratio of glucosylceramide to ceramide with SC ultrastructural disorders, for example, the presence of unprocessed lamellar bodies all through the SC [23].

Niemann-Pick disease is an autosomal recessive lysosomal storage disorder, in which two types can be distinguished: 1) acid sphingomyelinase-deficient Niemann-Pick disease with mutation in the SMPD1 gene (types A and B and intermediate forms) and 2) Niemann-Pick disease type C, also with type D, caused by mutations in either the NPC1 or NPC2 gene [81]. A topical application of acid sphingomyelinase inhibitor on the skin of mice significantly increased the level of sphingomyelin and decreased the level of ceramides, however, this observation was not significant. The application of acid sphingomyelinase inhibitors significantly delayed barrier recovery after acute barrier disruption, which significantly normalized after the co-application of ceramides [24]. Schmuth et al. [24] reported that patients with the intermediate form of Niemann-Pick disease had significantly delayed skin barrier recovery after tape stripping.

Yamamoto et al. [11] reported that the SC of patients with mild and moderate acne vulgaris contained a significantly lower percentage ratio of total ceramides and sphingosine with concomitant significant elevation of TEWL in comparison to healthy controls. In vitro studies revealed that phytosphingosine inhibited the growth of Gram-positive and Gram-negative bacteria, including Propionibacterium acnes, and in vivo investigations confirmed its antimicrobial properties through a significant reduction of total microbial count on unwashed hands. The randomized, half-face clinical trial in patients with acne vulgaris revealed that a treatment of benzoyl peroxide with phytosphingosine further reduced the number of comedones, papules and pustules in comparison to the application of benzoyl peroxide alone [82].

Recently, Jungersted et al. [10] reported that there is no statistically significant difference in stratum corneum ceramide profiles or in the ceramide/cholesterol ratio between two groups of patients with different etiology of hand eczema: exogenous (allergic/irritant) and endogenous (hyperkeratotic).

## Conclusions

Sphingolipids, which have biological and structural functions in epidermis, are importantly involved in the maintenance of the skin barrier function and regulate cellular processes such as proliferation, differentiation and apoptosis of keratinocytes. As many dermatologic diseases, including psoriasis, atopic dermatitis and ichthyoses, are associated with altered composition and metabolism of epidermal sphingolipids, more studies precisely determining the role of sphingolipids in the pathogenesis of skin disorders are required to develop novel pharmacological treatment opportunities.

## Abbreviations

ABCA12: ATP-binding cassette transporter A12
AD: atopic dermatitis
ASK1: apoptosis signal-regulating kinase 1
CER: ceramide
CER[EOH]: ceramide containing esterified ω-hydroxy fatty acid with 6-hydroxysphingosine
CER[EOP]: ceramide containing esterified ω-hydroxy fatty acid with phytosphingosine
ELOVL: elongase of long-chain fatty acids
FTY720: nonselective S1P receptor agonist
GM-CSF: granulocyte-macrophage colony-stimulating factor
IFN-γ: interferon gamma
IL: interleukin
JNK: c-jun N-terminal kinase
LOX: lipoxygenase
PASI: Psoriasis Area and Severity Index
PKB: protein kinase B
PKC-α: protein kinase C-alpha
S1P: sphingosine-1-phosphate
SC: stratum corneum
SPT: serine palmitoyltransferase
TEWL: transepidermal water loss
TNF-α: tumor necrosis factor alpha
UGCG: UDP-glucose ceramide glucosyltransferase
